# Extracorporeal Cardiopulmonary Resuscitation due to Left Atrial Myxoma With Severe Pulmonary Hypertension: A Challenging Combination

**DOI:** 10.1016/j.jaccas.2026.106957

**Published:** 2026-02-12

**Authors:** Julio Vázquez Reguera, Pedro Peña Ortega, María San Martín Bragado, Marta Caballero Belloch, Elena Fuente González, Adrián Torres Clares, Mario Galván Ruiz, Beatriz Aguiar Bermúdez

**Affiliations:** a Department of Cardiology, Hospital Universitario Dr. Negrín, Las Palmas de Gran Canaria, Spain; b Department of Intensive Care Medicine, Hospital Universitario Dr. Negrín, Las Palmas de Gran Canaria, Spain; c Department of Cardiac Surgery, Hospital Universitario Dr. Negrín, Las Palmas de Gran Canaria, Spain

**Keywords:** acute heart failure, cardiac assist devices, hemodynamics, pulmonary hypertension, right ventricle

## Abstract

**Background:**

Myxomas are the most common primary cardiac tumors. Presentation as sudden cardiac death is uncommon. Management of patients on circulatory support with severe pulmonary hypertension (PH) is challenging.

**Case Summary:**

A 55-year-old woman was diagnosed with a giant left atrial myxoma. She experienced cardiac arrest requiring extracorporeal cardiopulmonary resuscitation (ECPR). Management of combined pre- and postcapillary PH was the key to the weaning of circulatory support.

**Discussion:**

To our knowledge, this is the first reported case of ECPR owing to an atrial myxoma. It suggests that ECPR may be considered as a bridge to surgery in sudden cardiac death in atrial myxoma, although precapillary PH may complicate weaning from circulatory support.

**Take-Home Messages:**

ECPR can be a lifesaving strategy in atrial myxoma. Management with circulatory support can be challenging owing to precapillary PH after tumor resection. Pulmonary vasodilators can play a key role in these patients.

## Visual Summary

**Figure:**
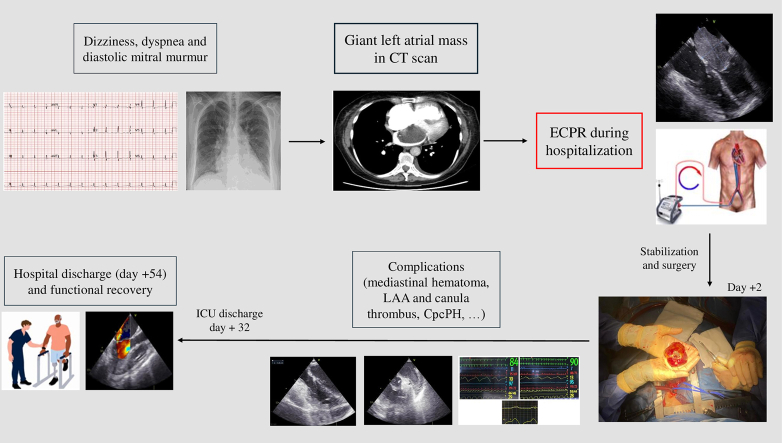

## History of Presentation

A 55-year-old woman presented to a private clinic with dizziness and dyspnea for several months. Physical examination revealed pulmonary crackles, severe lower limb edema, and a diastolic mitral murmur.

## Past Medical History

The patient was a smoker (never evaluated with pulmonary function testing), had hypertension, and was in treatment with levothyroxine for extirpation of the thyroid gland because of follicular cancer. There was no relevant family medical history.

## Differential Diagnosis

After detection of the atrial mass, the most likely diagnosis was an atrial myxoma, the most common primary cardiac tumor in adults. It typically arises from the interatrial septum, may exhibit microcalcifications, and is frequently associated with obstructive symptoms that mimic mitral valve disease. Other possibilities included a left atrial thrombus, particularly in the context of atrial fibrillation, or other primary cardiac tumors such as papillary fibroelastoma or lipoma, which are less frequent and usually smaller. Secondary cardiac metastases could also present as an atrial mass.

## Investigations

**Figure 1:**
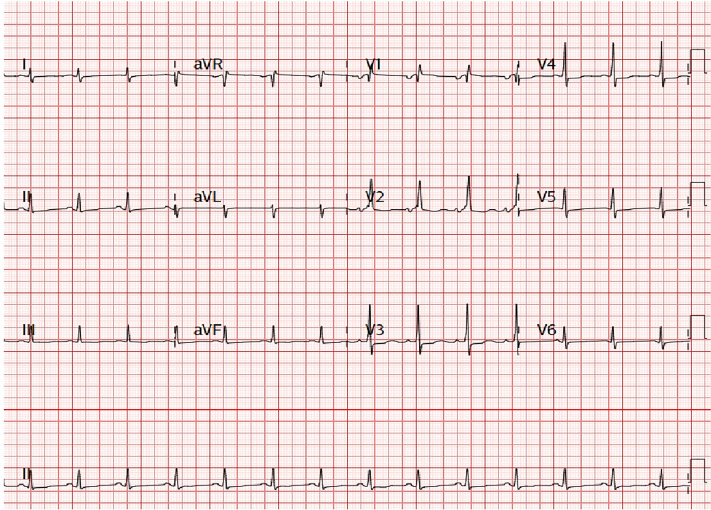
Electrocardiogram The electrocardiogram showed sinus rhythm and right bundle branch block.

**Figure 2:**
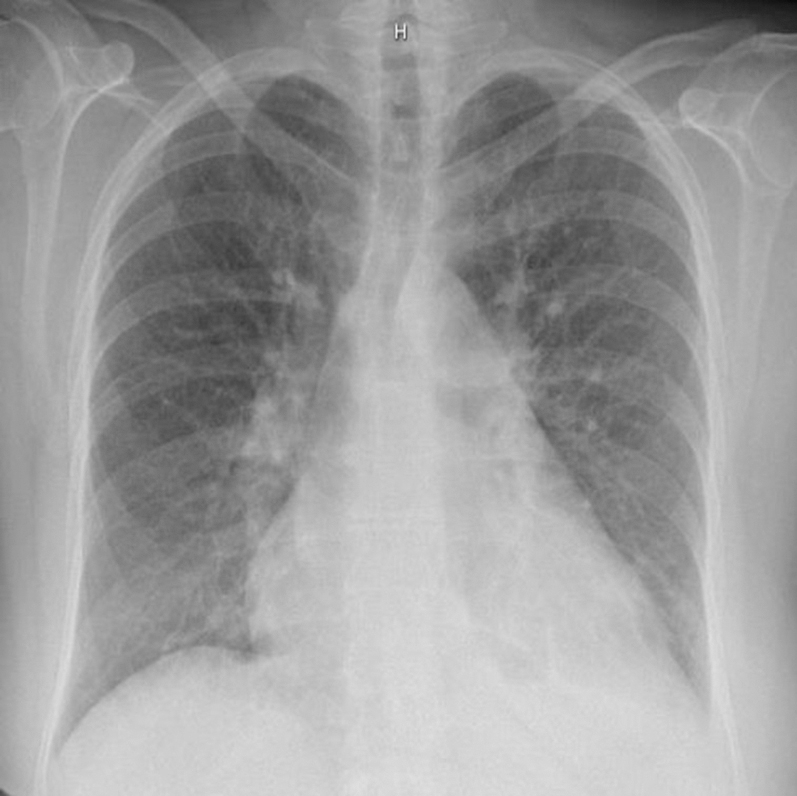
Initial Chest X-Ray The chest x-ray demonstrated cardiomegaly with mild pulmonary vascular redistribution.

**Figure 3:**
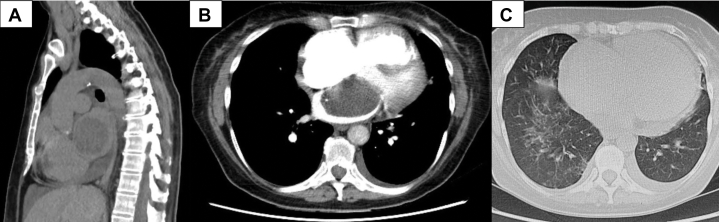
Computed Tomography Angiography (A and B) Computed tomography angiography showed a giant left atrial mass and light pericardial effusion. (C) Signs of heart failure were also observed.

All investigations were performed at the private clinic. Electrocardiogram showed sinus rhythm and right bundle branch block (Figure 1). Chest x-ray demonstrated cardiomegaly (Figure 2). Initially, given poor response to depletive therapy, computed tomography angiography was performed to exclude pulmonary embolism, revealing a left atrial mass, a mild pericardial effusion, and findings consistent with heart failure (Figure 3).

**Figure 4:**
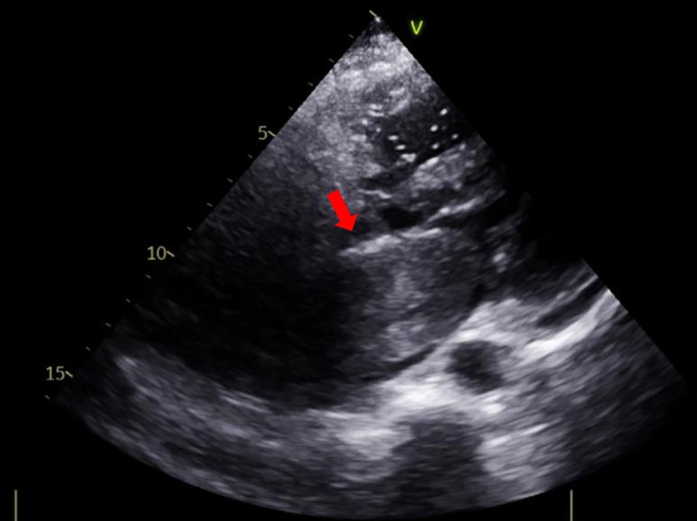
Baseline Echocardiogram Initial echocardiogram revealed a left atrial mass attached to the interatrial septum, protruding into the left ventricle (red arrow).

The initial echocardiogram revealed a giant left atrial mass attached to the interatrial septum, with internal microcalcifications, protruding into the left ventricle and causing marked mitral obstruction (Figure 4, Video 1). There was severe left atrial enlargement, preserved left ventricular ejection fraction, and evidence of right ventricular dysfunction with severe pulmonary hypertension (PH) (estimated pulmonary artery systolic pressure: 100 mm Hg).

## Management

**Figure 5:**
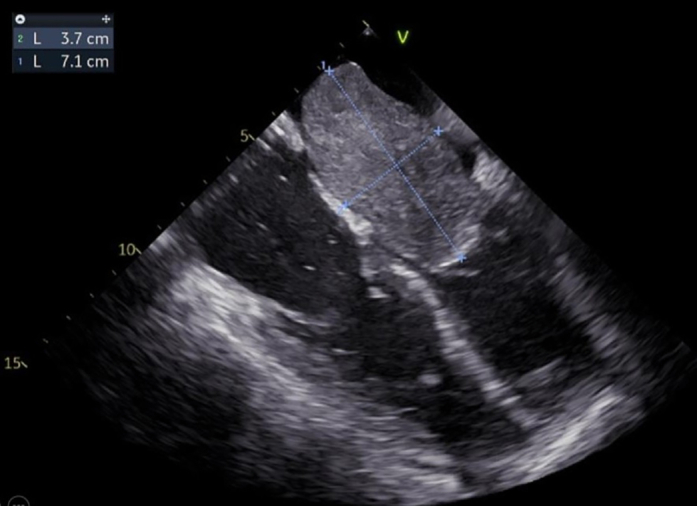
Transesophageal Echocardiography Transesophageal echocardiography showed a 7.1 × 3.7 cm interatrial septal mass occupying the entire left atrium.

The patient was transferred to a referral center for surgical evaluation. The day after admission, she developed worsening dyspnea and perioral cyanosis after intravenous furosemide, progressing to severe bradycardia and pulseless electrical activity arrest. Owing to refractoriness, bedside femorofemoral venoarterial extracorporeal membrane oxygenation (VA-ECMO) was implanted under transesophageal echocardiographic guidance, which revealed a 7.1 × 3.7 cm interatrial septal mass protruding into the left ventricle and occupying the entire left atrium (Figure 5, Videos 2 and 3). After 39 minutes of cardiac arrest, sinus rhythm and cardiac contractility were restored, with severe biventricular dysfunction and moderate pericardial effusion (Video 4).

Urgent surgery was deferred because of shock. During the first hours, the patient developed anemia and progressive thrombocytopenia secondary to a right lateral cervical hematoma due to right internal jugular central venous catheter placement. Computed tomography demonstrated an active venous bleeding focus in the sternoclavicular fossa, requiring urgent vascular surgical intervention. Also, a large thrombus was seen in the venous canula, resolved with anticoagulation (Video 5).

**Figure 6:**
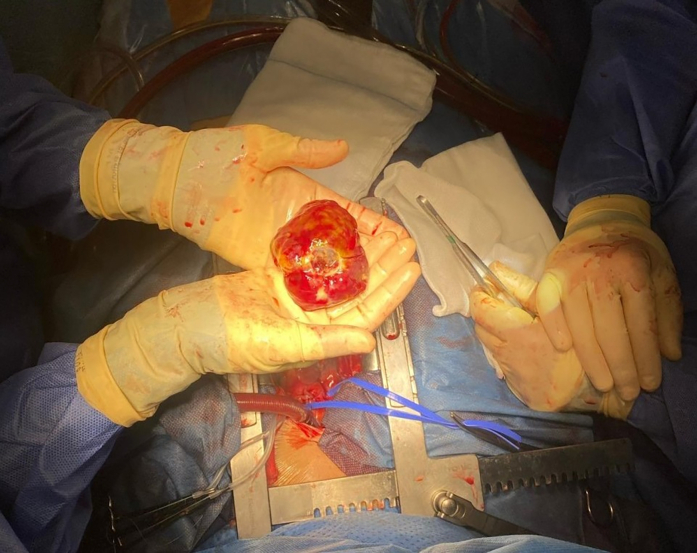
Surgical Resection Surgical resection of the atrial mass through a right atrial approach.

**Figure 7:**
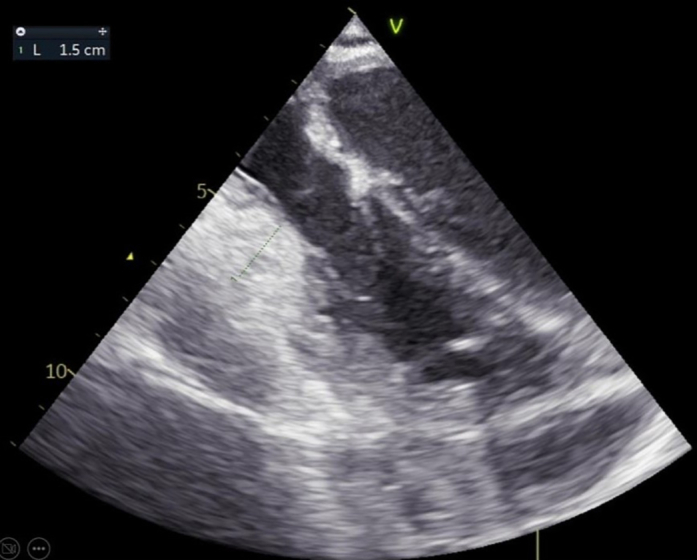
Mediastinal Hematoma Transesophageal echocardiography showed a mediastinal hematoma, which required surgical evacuation.

Hemodynamic stabilization, with normalization of lactate and metabolic parameters, reduction in vasopressor support, and a favorable neurological profile, led to surgical resection of the mass on day 2 via a right atrial approach (Figure 6). The patient returned from the operating room on VA-ECMO. Initial postoperative recovery was satisfactory. However, on day 4, she developed hemodynamic deterioration with increased fluid and norepinephrine requirements along with rapid ventricular response atrial fibrillation. A thrombus was also identified in the left atrial appendage (Video 6). Transesophageal echocardiography suggested the presence of mediastinal hematoma (Figure 7, Video 7), which required surgical evacuation, resulting in clinical improvement.

**Figure 8:**
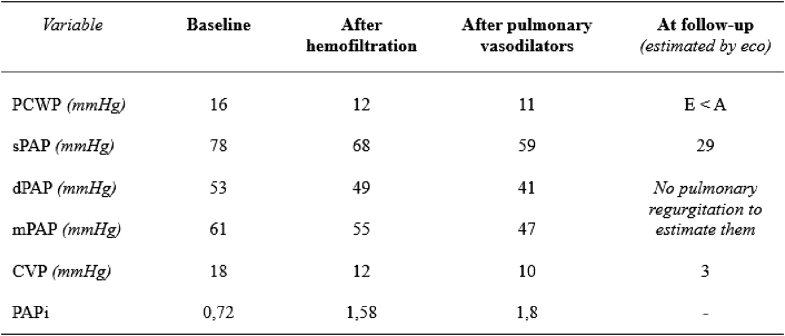
Evolution of Pulmonary Pressures and Pulmonary Artery Pulsatility Index All measurements (if not otherwise indicated) were taken using a Swan-Ganz catheter. CVP = central venous pressure; dPAP = diastolic pulmonary artery pressure; mPAP = mean pulmonary artery pressure; PAPi = pulmonary artery pulsatility index; PCWP = pulmonary capillary wedge pressure; sPAP = systolic pulmonary artery pressure.

**Figure 9:**
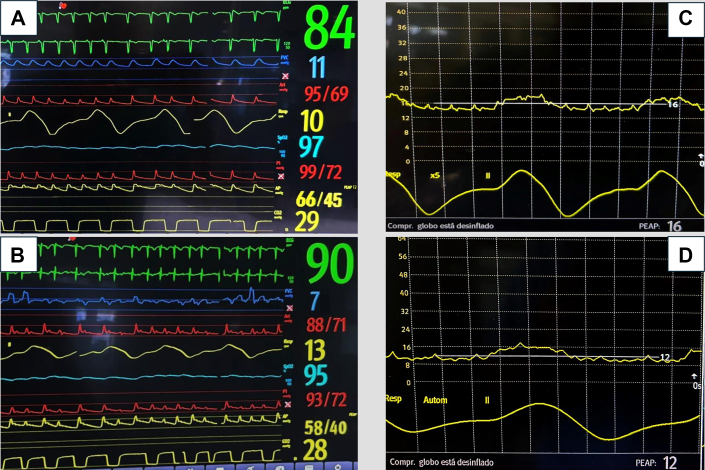
Evolution of Invasive Hemodynamics Evolution of pulmonary artery pressures (A) after volume optimization and (B) after pulmonary vasodilator administration. (C) PCWP was slightly elevated before volume optimization (D) and remained within normal range afterward. PCWP = pulmonary capillary wedge pressure.

On subsequent days, a pulmonary artery catheter was inserted given unfavorable clinical progression. The patient's pulmonary capillary wedge pressure (PCWP) was slightly elevated (≈16 mm Hg) after the mass resection. After volume optimization, she persistently exhibited elevated pulmonary pressures despite normal PCWP, with a high transpulmonary gradient (≈35 mm Hg), suggesting a precapillary component. There were no significant findings on chest x-ray. VA-ECMO flow reductions were poorly tolerated, resulting in right ventricular deterioration and increased inotropic requirements. Milrinone was initiated to achieve pulmonary arterial vasodilation and enhance right ventricular contractility, but it was not tolerated owing to recovery of left ventricular systolic function, resulting in a “kissing walls” phenomenon and severe hypotension. A right ventricular assist device was considered if medical therapy failed. With normalized PCWP and severe precapillary PH, inhaled pulmonary vasodilators (nitric oxide) and intravenous sildenafil were initiated, resulting in hemodynamic improvement with reductions in pulmonary artery pressures (Figures 8 and 9). This led to an improvement in right ventricular function (pulmonary artery pulsatility index improved from 0.72 to 1.8) and successful VA-ECMO weaning on day 13 without complications.

The patient's course after decannulation was favorable. She required prolonged ventilatory weaning owing to critical illness neuropathy and severe sarcopenia, requiring tracheostomy and rehabilitation. Her last hospitalization echocardiography showed preserved left ventricular ejection fraction with right ventricular dysfunction, moderate tricuspid regurgitation (estimated systolic pulmonary artery pressure: 50 mm Hg), and mild mitral regurgitation. She refused to undergo right heart catheterization for further evaluation of PH. She was discharged from the intensive care unit on day 32 and from the hospital on day 54, on sildenafil 10 mg every 8 hours, dapagliflozin 10 mg once daily, and spironolactone 25 mg once daily.

## Outcome and Follow-Up

**Figure 10:**
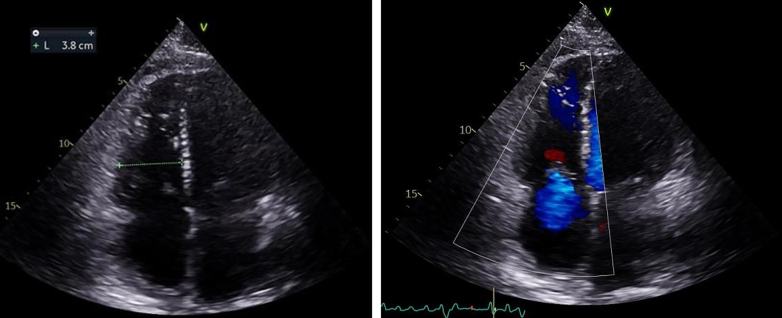
Follow-Up Echocardiography Follow-up echocardiography shows preserved left and right ventricular function, no mitral regurgitation, and moderate tricuspid regurgitation.

At the 1-year follow-up, the patient was ambulatory at NYHA functional class I status, walking with a walker. She was in sinus rhythm, with complete recovery of renal function and with a pro–B-type natriuretic peptide level of 207 ng/dL. Echocardiography demonstrated preserved left and right ventricular function, no mitral regurgitation, and moderate tricuspid regurgitation, with an estimated systolic pulmonary artery pressure of 29 mm Hg (at the 6-month follow-up the pulmonary artery systolic pressure was 35 mm Hg, and sildenafil had been discontinued) (Figure 10, Video 8). Pulmonary function tests were normal. Current therapy included spironolactone, dapagliflozin, and antihypertensive medication. No more hospitalizations had been required.

## Discussion

Primary cardiac tumors are rare, with myxomas being the most common. The majority of myxomas are located in the left atrium and originate from the fossa ovalis. Other locations are possible, as anterior leaflet of the mitral valve or right ventricle, which are rare. The majority present with heart failure symptoms such as dyspnea, orthopnea, and elevated venous pressure, as in our patient. Other major clinical manifestations include thromboembolic episodes, mostly resembling transient ischemic attack, and/or systemic embolisms. Most of these clinical forms are not life threatening, but they can be potentially fatal with the continuous growth of the tumor, leading even to sudden cardiac death.

The literature consistently supports urgent surgery once diagnosis is confirmed, regardless of age, given the risk of further hemodynamic compromise and recurrent arrest.^1^ Preoperative stabilization should include management of heart failure and pulmonary congestion, correction of metabolic derangements, and continuous monitoring for arrhythmias or embolic events. In case of refractory cardiac arrest and shock, temporary mechanical circulatory support with VA-ECMO seems to be appropriate as a bridge to definitive surgery, although we have not found any cases of extracorporeal cardiopulmonary resuscitation (ECPR) in the setting of cardiac arrest due to an atrial myxoma. We support allowing time for stabilization and organ recovery prior to resection after cardiac arrest to improve surgery outcomes.^2^^,^^3^

A biatrial approach is typically used during surgery to determine the tumor origin, inspect all 4 cardiac chambers for tumor fragments, and assess mitral or tricuspid valve involvement. Complete pedicle resection of the tumor stalk and its attachment is recommended to prevent recurrence, particularly when originating from the fossa ovalis.^4^

Another key aspect of this case was PH management to facilitate VA-ECMO weaning. Combined pre- and postcapillary PH has been described in long-standing atrial myxoma.^5^^,^^6^ With the growing use of ECPR, clinicians are increasingly managing patients on extracorporeal support who would likely have been deemed unsuitable under elective conditions, particularly those with severe precapillary PH. This scenario presents significant challenges for circulatory support management and weaning. Pulmonary vasodilators (mainly inhaled nitric oxide/iloprost or intravenous epoprostenol/sildenafil) have limited evidence in cardiogenic shock, with no proven benefit in patients with combined pre- and postcapillary PH.^7^^,^^8^ In our case, after mass resection, the patient remained on VA-ECMO with predominantly precapillary PH, an exceptional scenario. Pulmonary vasodilators may play a key role in this scenario, reducing right ventricular afterload, thereby enabling tolerance of the increased preload associated with VA-ECMO withdrawal and facilitating successful weaning. Milrinone or levosimendan may be also useful in this context given their combined vasodilatory and inotropic effects. Right ventricular assist devices were considered if medical therapy had been ineffective. However, the risk of pulmonary complications due to pulmonary blood flow increase in a patient with precapillary PH was high, and such devices may not be beneficial in this group of patients.^9^^,^^10^ At follow-up, the patient exhibited no significant PH, suggesting reversibility of the precapillary component and confirming that it was secondary to long-standing high left-heart pressures caused by the atrial myxoma. To our knowledge, no other cases have reported reversibility of precapillary PH after atrial myxoma resection.

**Visual Summary:**
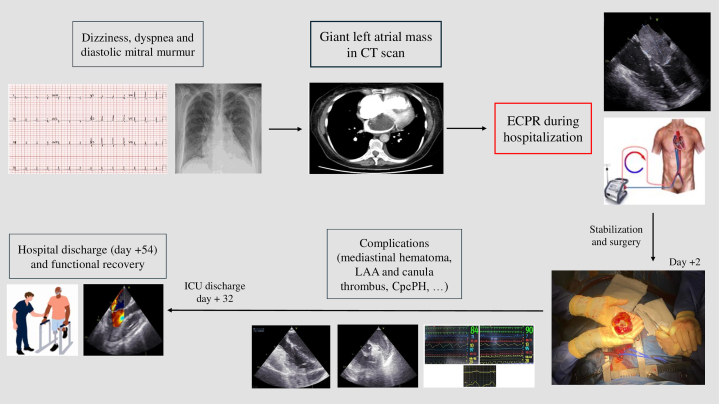
Summary of Case Presentation CpcPH = combined pre- and postcapillary pulmonary hypertension; CT = computed tomography; ECPR = extracorporeal cardiopulmonary resuscitation; ICU = intensive care unit; LAA = left atrial appendage.

## Conclusions

This is to our knowledge the first reported case of ECPR performed for cardiac arrest secondary to an atrial myxoma. Management with temporary mechanical circulatory support remains challenging and is supported by limited evidence, especially in those patients with severe precapillary PH. Pulmonary vasodilators may play a pivotal role in facilitating successful weaning from circulatory support.

## Funding Support and Author Disclosures

The authors have reported that they have no relationships relevant to the contents of this paper to disclose.

**Take-Home Messages**

• Extracorporeal cardiopulmonary resuscitation can be a lifesaving strategy in atrial myxoma. Management with circulatory support can be challenging owing to precapillary PH after tumor resection.
• Pulmonary vasodilators can play a key role in these patients.

## Appendix

Video 1 Baseline Echocardiogram Initial echocardiogram revealed a left atrial mass attached to the interatrial septum, protruding into the left ventricle.

Video 2 Transesophageal Echocardiography Transesophageal echocardiography showing interatrial septal mass occupying the entire left atrium.

Video 3 Transesophageal Echocardiography Transesophageal echocardiography showing interatrial septal mass occupying the entire left atrium.

Video 4 Echocardiogram After Cardiac Arrest After restoration of sinus rhythm and myocardial contractility, severe biventricular dysfunction and moderate pericardial effusion were observed.

Video 5 Canula Thrombus A large thrombus was seen in the venous canula, resolved with anticoagulation.

Video 6 LAA Thrombus LAA thrombus due to atrial fibrillation was observed and was resolved with anticoagulation. LAA = left atrial appendage.

Video 7 Mediastinal Hematoma Transesophageal echocardiography showed a mediastinal hematoma, which required surgical evacuation.

Video 8 Follow-Up Echocardiography Follow-up echocardiography shows preserved left and right ventricular function, no mitral regurgitation, and moderate tricuspid regurgitation.
